# Recent advances in (therapeutic protein) drug development

**DOI:** 10.12688/f1000research.9970.1

**Published:** 2017-02-07

**Authors:** H.A. Daniel Lagassé, Aikaterini Alexaki, Vijaya L. Simhadri, Nobuko H. Katagiri, Wojciech Jankowski, Zuben E. Sauna, Chava Kimchi-Sarfaty

**Affiliations:** 1 Hemostasis Branch, Division of Plasma Protein Therapeutics, Office of Tissues and Advanced Therapies, Center for Biologics Evaluation and Research, U.S. Food and Drug Administration, Silver Spring, MD, USA

**Keywords:** therapeutic protein drugs, protein therapeutics, cancer therapeutics, biosimilar, recombinant DNA-derived therapeutic proteins

## Abstract

Therapeutic protein drugs are an important class of medicines serving patients most in need of novel therapies. Recently approved recombinant protein therapeutics have been developed to treat a wide variety of clinical indications, including cancers, autoimmunity/inflammation, exposure to infectious agents, and genetic disorders. The latest advances in protein-engineering technologies have allowed drug developers and manufacturers to fine-tune and exploit desirable functional characteristics of proteins of interest while maintaining (and in some cases enhancing) product safety or efficacy or both. In this review, we highlight the emerging trends and approaches in protein drug development by using examples of therapeutic proteins approved by the U.S. Food and Drug Administration over the previous five years (2011–2016, namely January 1, 2011, through August 31, 2016).

## Protein engineering

**Figure 1.**
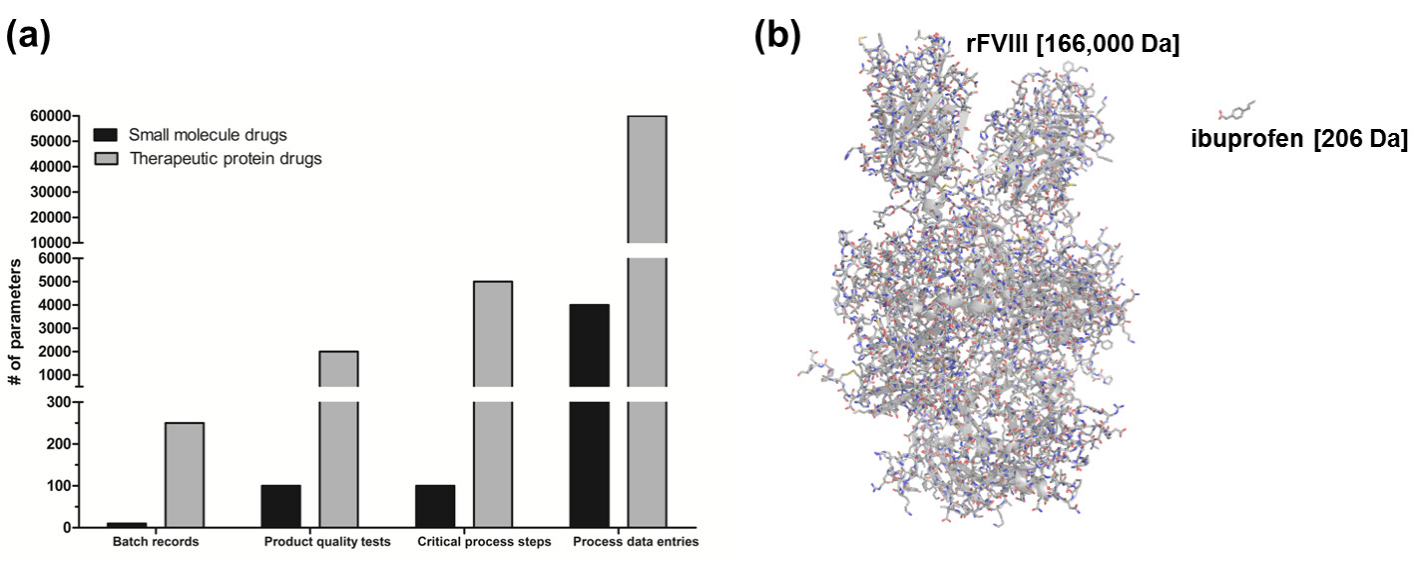
Complexity of therapeutic protein drugs. ( **a**) Graphical representation of the complexity of the manufacture of a therapeutic protein drug compared with a small-molecule drug. The number of batch records, product quality tests, critical process steps, and process data entries associated with small-molecule drugs (black) and therapeutic protein drugs (grey) as bars ^4^. ( **b**) Illustration depicting the differences in size and complexity of a protein therapeutic (recombinant (r) analogue of human coagulation factor VIII (FVIII); Novoeight, Novo Nordisk; molecular weight = 166,000 Da) and a small-molecule drug (ibuprofen; molecular weight = 206 Da) by molecular model.

The manufacturing and production of therapeutic proteins are highly complex processes ^1– 3^. For example, a typical protein drug may include in excess of 5,000 critical process steps, many times greater than the number required for manufacturing a small-molecule drug ^4^ ( Figure 1a).

Similarly, protein therapeutics, which include monoclonal antibodies as well as large or fusion proteins, can be orders-of-magnitude larger in size than small-molecule drugs, having molecular weights exceeding 100 kDa ( Figure 1b). In addition, protein therapeutics exhibit complex secondary and tertiary structures that must be maintained. Protein therapeutics cannot be completely synthesized by chemical processes and have to be manufactured in living cells or organisms; consequently, the choices of the cell line, species origin, and culture conditions all affect the final product characteristics ^5– 7^. Moreover, most biologically active proteins require post-translational modifications that can be compromised when heterologous expression systems are used. Additionally, as the products are synthesized by cells or organisms, complex purification processes are involved. Furthermore, viral clearance processes such as removal of virus particles by using filters or resins, as well as inactivation steps by using low pH or detergents, are implemented to prevent the serious safety issue of viral contamination of protein drug substances ^8^. Given the complexity of therapeutic proteins with respect to their large molecular size, post-translational modifications, and the variety of biological materials involved in their manufacturing process, the ability to enhance particular functional attributes while maintaining product safety and efficacy achieved through protein-engineering strategies is highly desirable.

While the integration of novel strategies and approaches to modify protein drug products is not a trivial matter ^9^, the potential therapeutic advantages have driven the increased use of such strategies during drug development. A number of protein-engineering platform technologies are currently in use to increase the circulating half-life, targeting, and functionality of novel therapeutic protein drugs as well as to increase production yield and product purity ( Table 1) ^5– 7, 10– 12^. For example, protein conjugation and derivatization approaches, including Fc-fusion ^13, 14^, albumin-fusion ^15^, and PEGylation ^16^, are currently being used to extend a drug’s circulating half-life ^17^. Longer *in vivo* half-lives are of particular importance to patients undergoing factor/enzyme/hormone replacement therapy, in which frequent dosing regimens can result in substantial negative impacts on patient well-being in terms of ease of administration and compliance, especially in young children ^18^. Protein-engineering approaches have also been employed to target drugs through the addition of signaling peptides or the generation of antibody-drug conjugates ^19^, thereby limiting toxicity and increasing drug efficacy. Additionally, exploiting particular functional characteristics of a protein drug can be accomplished through protein engineering. For example, influencing a protein’s glycosylation pattern through engineering strategies can impact the protein’s receptor-binding properties and overall effector function ^20, 21^. In Table 1, we have highlighted a few examples of the many technological innovations and protein-engineering platform technologies incorporated by recently approved therapeutic proteins.

**Table 1.** Protein-engineering platform technologies.

Platform technology	Example of U.S. Food and Drug Administration-approved therapeutic protein
Protein production technologies	
Production of proteins in transgenic animals ^46^	C1 esterase inhibitor (Ruconest) produced in transgenic rabbit milk ^47^
Production of proteins in transgenic plants ^48^	Human glucocerebrosidase (Elelyso) produced in carrot root cells ^49, 50^
Rational protein structure/function technologies	
Glyco-engineering ^20, 21^	Humanized anti-CD20 monoclonal antibody (Gazyva) ^51^
Fc fusion ^13, 14^	VEGFR Fc-fusion (Eylea)
	CTLA-4 Fc-fusion (Nulojix)
	Glucagon-like peptide- 1 receptor agonist Fc-fusion (Trulicity)
	VEGFR Fc-fusion (Zaltrap)
	Recombinant factor IX Fc fusion (Alprolix) ^52^
	Recombinant factor VIII Fc-fusion (Eloctate) ^53, 54^
Albumin fusion ^15^	GLP-1 receptor agonist-albumin fusion (Tanzeum)
	Recombinant factor IX albumin fusion (Idelvion)
PEGylation ^55^	PEGylated IFNβ-1a (Plegridy)
	Recombinant factor VIII PEGylated (Adynovate)
Antibody-drug conjugates ^19^	Humanized anti-HER2/neu conjugated to emtansine (Kadcyla)
	Mouse/human chimeric anti-CD30 (Adcetris)
mAb humanization/chimerism	Humanized mAbs
	Anti-human epidermal growth factor receptor 2 (HER2) (Perjeta)
	Anti-HER2/neu conjugated to emtansine (Kadcyla)
	Anti-IL-6 receptor (Actemra)
	Anti-CD20 (obinutuzumab; Gazyva)
	Anti-integrin a4b7 (LPAM-1) (Entyvio)
	Anti-PD-1 (Keytruda)
	Anti-dabigatran (Praxbind)
	Anti-IL-5 (Nucala)
	Anti-CD319 (SLAMF7) (Empliciti)
	Anti-IL-17a (Taltz)
	Anti-IL-5 (Cinqair)
	Anti-PD-L1 (Tecentriq)
	Anti-CD25 (Zinbryta)

	Mouse/human chimeric mAbs
	Anti-CD30 (Adcetris)
	Anti-IL-6 (Sylvant)
	Anti-GD2 (Unituxin)
	Anti- *Bacillus anthracis* (Anthim)
	Anti-TNFα (Inflectra)

Listing of commonly used protein-engineering platform technologies and examples of U.S. Food and Drug Administration-approved therapeutic proteins (2011–2016, namely January 1, 2011, through August 31, 2016) that employ each strategy. CD, cluster of differentiation; CTLA-4, cytotoxic T-lymphocyte-associated protein 4; Fc, fragment crystallizable; GD2, disialoganglioside; GLP-1, glucagon-like peptide-1; HER2, human epidermal growth factor receptor 2; IFNb, interferon beta; IL, interleukin; LPAM-1, lymphocyte Peyer’s Patch adhesion molecule; mAb, monoclonal antibody; PD-1, programmed death receptor-1; PD-L1, programmed death-ligand 1; PEG, polyethylene glycol; SLAMF7, SLAM family member 7; TNFα, tumor necrosis factor alpha; VEGFR, vascular endothelial growth factor receptor.

## Overview of recently approved protein therapeutics (2011–2016*)

**Figure 2.**
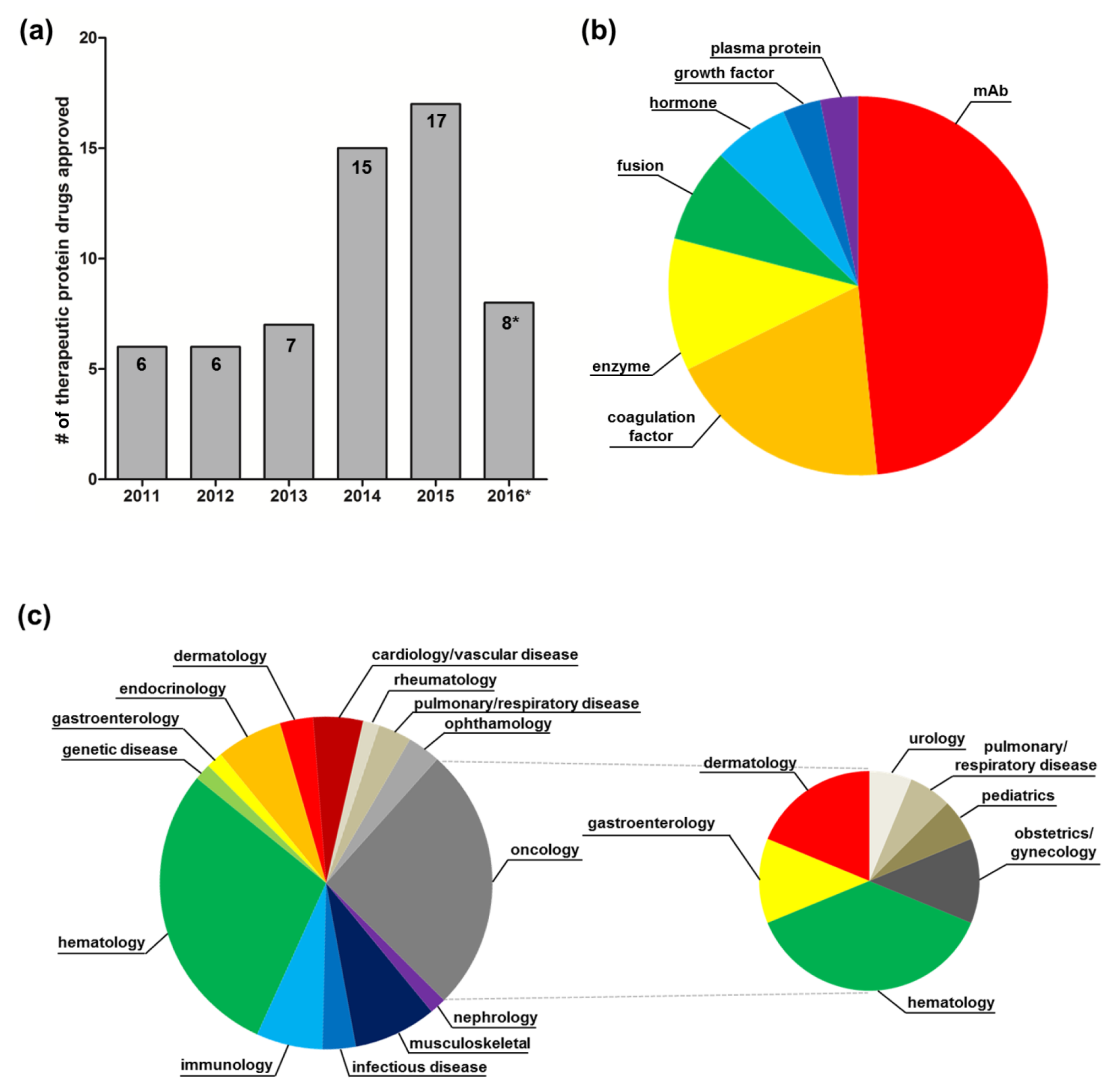
U.S. Food and Drug Administration (FDA)-approved therapeutic proteins (2011–2016*). ( **a**) Bar graph showing the number of therapeutic protein FDA approvals by year (2011–2016*). ( **b**) Pie chart showing the distribution of FDA-approved therapeutic proteins (2011–2016*) by drug class. ( **c**) (Left) Pie chart showing the distribution of FDA-approved therapeutic proteins (2011–2016*) by therapeutic area. (Right) Pie chart showing the distribution of secondary therapeutic area for oncology drugs. *January 1, 2011, through August 31, 2016.

Since 2011, the U.S. Food and Drug Administration Center for Drug Evaluation and Review (CDER) and the Center for Biologics Evaluation and Review (CBER) combined have approved 62 recombinant therapeutic proteins (*January 1, 2011, through August 31, 2016; “Purple Book” list of licensed biological products, including biosimilar and interchangeable biological products ^22^) ( Figure 2a). Of these 62 therapeutic proteins, almost half (48%) were monoclonal antibodies (for this analysis, we included antibody-drug conjugates and antibody fragment antigen binding in this group). Coagulation factors were the next largest class (19%) of approved protein drugs over this time period. Replacement enzymes comprised 11% of all approvals. Remaining approvals (22%) were divided among fusion proteins, hormones, growth factors, and plasma proteins ( Figure 2b). These U.S. Food and Drug Administration (FDA)-approved therapeutic proteins are indicated for a wide variety of therapeutic areas. Over half of the approved therapeutic proteins were indicated for oncology (26%) and hematology (29%), whereas the remaining 45% had primary indications in cardiology/vascular disease (5%), dermatology (3%), endocrinology (6%), gastroenterology (2%), genetic disease (2%), immunology (6%), infectious diseases (3%), musculoskeletal (8%), nephrology (2%), ophthalmology (3%), pulmonary/respiratory disease (3%), and rheumatology (2%) ( Figure 2c, left). Of the 16 oncology drugs, six were approved to treat hematologic malignancies, whereas the remaining therapeutics were indicated for dermatology (3), gastroenterology (2), obstetrics/gynecology (2), pediatrics (1), pulmonary/respiratory disease (1), and urology (1) ( Figure 2c, right). For a complete listing of the approved products, see Table 2. It is evident that recently approved therapeutic proteins serve a wide spectrum of patient populations and are of great benefit to public health.

**Table 2.** U.S. Food and Drug Administration-approved protein therapeutics (2011–2016).

CDER approved protein therapeutics [2011–2016*]			
# Approval Date	Drug [Market Name; Sponsor]	Class [Description]	Therapeutic Area [General Indication]
**1** 3/9/2011	belimumab [ Benlysta; Human Genome Sciences]	mAb [human anti-B-cell activating factor (BAFF)]	immunology [autoimmunity (lupus)]
**2** 3/25/2011	ipilimumab [ Yervoy; Bristol Myers Squibb]	mAb [human anti-CTLA-4]	dermatology/oncology [cancer (melanoma)]
**3** 6/15/2011	belatacept [ Nulojix; Bristol Myers Squibb]	Fc fusion [CTLA-4 Fc-fusion]	immunology/nephrology [transplant rejection (kidney)]
**4** 8/19/2011	brentuximab vedotin [ Adcetris; Seattle Genetics]	antibody-drug conjugate [mouse/human chimeric anti- CD30]	hematology/oncology [cancer (lymphoma)]
**5** 11/18/2011	afilbercept [ Eylea; Regeneron Pharmaceuticals]	Fc fusion [VEGFR Fc-fusion]	ophthalmology [macular degeneration]
**6** 11/18/2011	asparaginase erwinia chrysanthemi [ Erwinaze; Jazz Pharmaceuticals]	enzyme [asparaginase erwinia chrysanthemi]	hematology/oncology [cancer (leukemia)]
**7** 1/17/2012	glucarpidase [ Voraxaze; BTG International]	enzyme [glucarpidase]	nephrology [kidney failure]
**8** 5/1/2012	taliglucerase alfa [ Elelyso; Pfizer]	enzyme [β-glucocerebrosidase]	endocrinology/gastroenterology [genetic disorder (Gaucher)]
**9** 6/8/2012	pertuzumab [ Perjeta; Genentech]	mAb [humanized anti-human epidermal growth factor receptor 2 (HER2)]	obstetrics, gynecology/oncology [cancer (breast)]
**10** 8/3/2012	ziv-afilbercept [ Zaltrap; Sanofi-Aventis U.S.]	Fc fusion [VEGFR Fc fusion]	gastroenterology/oncology [cancer (colorectal)]
**11** 8/29/2012	tbo-filgrastim [ Granix; Cephalon]	growth factor [G-CSF]	hematology/oncology [neutropenia]
**12** 10/17/2012	ocriplasmin [ Jetrea; ThromboGenics]	enzyme [ocriplasmin]	ophthalmology [eye condition (symptomatic vitreomacular adhesion)]
**13** 12/14/2012	raxibacumab [ raxibacumab; Human Genome Sciences]	mAb [human anti-anthrax protective antigen (PA)]	infections and infectious disease [infectious disease (inhalational anthrax)]
**14** 2/22/2013	ado-trastuzumab emtansine [ Kadcyla; Genentech]	antibody-drug conjugate [humanized anti-HER2/neu conjugated to emtansine]	obstetrics, gynecology/oncology [cancer (breast)]
**15** 7/18/2013	golimumab injection, for IV use [ Simponi Aria; Janssen Biotech]	mAb [human anti-TNFα]	musculoskeletal/rheumatology [autoimmunity (rheumatoid arthritis)]
**16** 10/21/2013	tocilizumab [ Actemra; Genentech]	mAb [humanized anti-IL-6 receptor]	musculoskeletal/rheumatology [autoimmunity (rheumatoid arthritis; juvenile idiopathic arthritis)]
**17** 11/1/2013	obinutuzumab [ Gazyva; Genentech]	mAb [humanized anti-CD20]	hematology/oncology [cancer (leukemia)]
**18** 2/14/2014	elosulfase alfa [ Vimizim; BioMarin Pharmaceutical]	enzyme [elosulfase alfa]	musculoskeletal/genetic disease [genetic disorder (Morquio A)]
**19** 2/24/2014	metreleptin [ Myalept; Amylin Pharmaceuticals]	hormone [metreleptin]	immunology [lipodystrophy]
**20** 4/15/2014	albiglutide [ Tanzeum; GlaxoSmithKline]	albumin fusion/hormone [glucagon-like peptide-1 dimer albumin fusion]	endocrinology [diabetes (type 2)]
**21** 4/21/2014	ramucirumab [ Cyramza; Eli Lilly and Company]	mAb [human anti-VEGFR2 (KDR)]	gastroenterology/oncology [cancer (stomach; gastroesophageal junction)]
**22** 4/23/2014	siltuximab [ Sylvant; Janssen Biotech]	mAb [mouse/human chimeric anti- IL-6]	hematology/immunology [multicentric Castleman's disease]
**23** 5/20/2014	vedolizumab [ Entyvio; Takeda Pharmaceuticals America]	mAb [humanized anti-integrin a4b7 (lymphocyte Peyer's Patch adhesion molecule; LPAM-1)]	gastroenterology/immunology [inflammatory (ulcerative colitis/Crohn's disease)]
**24** 8/15/2014	peginterferon beta-1a [ Plegridy; Biogen Idec]	cytokine [PEGylated IFNb-1b]	immunology/musculoskeletal [multiple sclerosis]
**25** 9/4/2014	pembrolizumab [ Keytruda; Merck Sharp & Dohme]	mAb [humanized anti-PD-1]	dermatology/oncology [cancer (melanoma)]
**26** 9/18/2014	dulaglutide [ Trulicity; Eli Lilly and Company]	Fc fusion [glucagon-like peptide-1 receptor agonist]	endocrinology [diabetes (type 2)]
**27** 12/3/2014	blintumomab [ Blincyto; Amgen]	mAb [mouse bispecific anti-CD19/ anti-CD3]	hematology/oncology [cancer (leukemia)]
**28** 12/22/2014	nivolumab [ Opdivo; Bristol Myers Squibb]	mAb [human anti-PD-1]	dermatology/oncology [cancer (melanoma)]
**29** 1/21/2015	secukinumab [ Cosentyx; Novartis Pharmaceuticals]	mAb [human anti-IL-17A]	dermatology/immunology [autoimmunity (plaque psoriasis)]
**30** 1/23/2015	parathyroid hormone [ Natpara; NPS Pharmaceuticals]	hormone [parathyroid hormone]	endocrinology/hematology [hypoparathyroidism]
**31** 3/6/2015	filgrastim-sndz [ Zarxio; Sandoz]	growth factor [G-CSF]	hematology/oncology [neutropenia]
**32** 3/10/2015	dinutuximab [ Unituxin; United Therapeutics]	mAb [mouse/human chimeric anti- GD2]	oncology/pediatrics/neonatalogy [cancer (neuroblastoma)]
**33** 7/24/2015	alirocumab [ Praluent; Sanofi-Aventis U.S.]	mAb [human anti-proprotein convertase substilisin/kexin type 9 (PCSK9)]	cardiology/vascular diseases [high cholesterol]
**34** 8/27/2015	evolocumab [ Repatha; Amgen]	mAb [human anti-proprotein convertase substilisin/kexin type 9 (PCSK9)]	cardiology/vascular diseases [high cholesterol]
**35** 10/16/2015	idarucizumab [ Praxbind; Boehringer Ingelheim Pharmaceuticals]	Fab [humanized anti-dabigatran]	hematology [anticoagulant reversal]
**36** 10/23/2015	asfotase-alfa [ Strensiq; Alexion Pharmaceuticals]	Fc fusion/enzyme [tissue non-specific alkaline phosphatase/Fc fusion/deca- asparatate (D10) peptide]	genetic disease/pediatrics/neonatalogy [genetic disorder (hypophosphatasia)]
**37** 11/4/2015	mepolizumab [ Nucala; GlaxoSmithKline]	mAb [humanized anti-IL-5]	pulmonary/respiratory disease [asthma]
**38** 11/16/2015	daratumumab [ Darzalex; Janssen Biotech]	mAb [human anti-CD38]	hematology/oncology [cancer (multiple myeloma)]
**39** 11/24/2015	necitumumab [ Portrazza; Eli Lilly and Company]	mAb [human anti-epidermal growth factor receptor (EGFR)]	pulmonary/respiratory disease/oncology [cancer (lung)]
**40** 11/30/2015	elotuzumab [ Empliciti; Bristol Myers Squibb]	mAb [humanized anti- CD319(SLAMF7)]	oncology [cancer (multiple myeloma)]
**41** 12/8/2015	sebelipase alfa [ Kanuma; Alexion Pharmaceuticals]	enzyme [lysosomal acid lipase]	cardiology/vascular diseases/genetic disease [lysosomal acid lipase deficiency]
**42** 3/18/2016	obiltoxaximab [ Anthim; Elusys Therapeutics]	mAb [mouse/human chimeric anti- *Bacillus anthracis*]	infections and infectious disease [infectious disease (inhalational anthrax)]
**43** 3/22/2016	ixekizumab [ Taltz; Eli Lilly and Company]	mAb [humanized anti-IL-17a]	dermatology/immunology [autoimmunity (plaque psoriasis)]
**44** 3/23/2016	reslizumab [ Cinqair; Teva Respiratory]	mAb [humanized anti-IL-5]	pulmonary/respiratory disease [asthma]
**45** 4/5/2016	infliximab-dyyb [ Inflectra; Celltrion]	mAb [mouse/human chimeric anti- TNFα]	musculoskeletal/rheumatology [inflammatory (Crohn's disease/ulcerative colitis/rheumatoid arthritis/ankylosing spondylitis/psoriatic arthritis/plaque psoriasis)]
**46** 5/18/2016	atezolizumab [ Tecentriq; Genentech]	mAb [humanized anti-PD-L1]	urology/oncology [cancer (bladder)]
**47** 5/27/2016	daclizumab [ Zinbryta; Biogen]	mAb [humanized anti-CD25]	musculoskeletal/neurology [multiple sclerosis]
**48** 8/30/2016	etanercept-szzs [ Erelzi; Sandoz]	Fc fusion [TNFR Fc-fusion]	rheumatology [inflammatory (rheumatoid arthritis/juvenile idiopathic arthritis/psoriatic arthritis/ankylosing spondylitis/plaque psoriasis)]
CBER approved protein therapeutics [2011–2016*]			
# Approval Date	Drug Name [Market Name; Sponsor]	Class Description	Therapeutic Area
**1** 6/26/2013	coagulation factor IX recombinant human [ Rixubis; Baxter Healthcare]	coagulation factor [recombinant factor IX]	hematology [hemophilia B]
**2** 10/15/2013	antihemophilic factor (recombinant) [ Novoeight; Novo Nordisk]	coagulation factor [recombinant factor VIII]	hematology [hemophilia A]
**3** 12/23/2013	coagulation factor XIII A- subunit (recombinant) [ Tretten; Novo Nordisk]	coagulation factor [recombinant factor XIII A subunit]	hematology [congenital factor XIII deficiency]
**4** 3/28/2014	coagulation factor IX (recombinant), Fc fusion protein [ Alprolix; Biogen]	Fc fusion/coagulation factor [recombinant factor IX Fc- fusion]	hematology [hemophilia B]
**5** 6/6/2014	antihemophilic factor (recombinant), Fc fusion protein [ Eloctate; Biogen]	Fc fusion/coagulation factor [recombinant factor VIII Fc- fusion]	hematology [hemophilia A]
**6** 7/16/2014	C1 esterase inhibitor recombinant [ Ruconest; Salix Pharmaceuticals]	plasma protein [recombinant C1 esterase inhibitor]	hematology [hereditary angioedema]
**7** 10/23/2014	antihemophilic factor porcine, B-domain truncated recombinant [ Obizur; Baxter Healthcare]	coagulation factor [recombinant factor VIII (porcine)]	hematology [hemophilia A]
**8** 4/29/2015	coagulation factor IX (recombinant) [ Ixinity; Cangene BioPharma]	coagulation factor [recombinant factor IX]	hematology [hemophilia B]
**9** 9/4/2015	antihemophilic factor (recombinant) [ Nuwiq; Octapharma USA]	coagulation factor [recombinant factor VIII]	hematology [hemophilia A]
**10** 11/13/2015	antihemophilic factor (recombinant) PEGylated [ Adynovate; Baxalta US]	coagulation factor [recombinant factor VIII PEGylated]	hematology [hemophilia A]
**11** 12/8/2015	von Willebrand factor (recombinant) [ Vonvendi; Baxalta US]	plasma protein [recombinant VWF]	hematology [von Willebrand disease]
**12** 3/4/2016	coagulation factor IX recombinant human [ Idelvion; CSL Behring Recombinant]	coagulation factor [recombinant factor IX albumin fusion]	hematology [hemophilia B]
**13** 3/16/2016	antihemophilic factor (recombinant) [ Kovaltry; Bayer HealthCare]	coagulation factor [recombinant factor VIII full- length]	hematology [hemophilia A]
**14** 5/25/2016	antihemophilic factor (recombinant) [ Afstyla; CSL Behring]	coagulation factor [recombinant factor VIII]	hematology [hemophilia A]

Comprehensive listing of all FDA-approved therapeutic proteins granted orphan designation upon original submission from January 1, 2011, through August 31, 2016, listed in chronological order of FDA approval. In addition, the class of protein, a brief description, and orphan designation are included. CD, cluster of differentiation; CTLA-4, cytotoxic T-lymphocyte-associated protein 4; Fab, fragment antigen binding; Fc, fragment crystallizable; GD2, disialoganglioside; IL, interleukin; mAb, monoclonal antibody; PD-1, programmed death receptor-1; VEGFR, vascular endothelial growth factor receptor.

## Pathways for the development of novel therapeutics

The rapid advances in biomedical science and technology to address unmet medical needs also require that regulatory agencies ensure that such products are safe and effective. Several new pathways have emerged or have been finalized since 2011 and are summarized below ( Table 3).

**Table 3.** Pathways for the development of novel therapeutics.

Pathway	Description and relevant U.S. Food and Drug Administration (FDA) guidances
Breakthrough therapy designation	“process designed to expedite the development and review of drugs that are intended to treat a serious condition and preliminary clinical evidence indicates that the drug may demonstrate substantial improvement over available therapy on a clinically significant endpoint(s)” ^56, 57^. Guidance for industry: Expedited Programs for Serious Conditions – Drugs and Biologics ^58^
Orphan designation	Rare disease or condition that affects 200,000 people or fewer per year in the U.S. ^59^ Guidance for industry: ( *Draft*) Rare Diseases: Common Issues in Drug Development ^60^
Biosimilar	‘an abbreviated licensure pathway for biological products that are demonstrated to be “biosimilar” to or “interchangeable” with an FDA-licensed biological product… a biological product may be demonstrated to be “biosimilar” if data show that, among other things, the product is “highly similar” to an already-approved biological product’ ^61^. Guidance for industry: Quality Considerations in Demonstrating Biosimilarity of a Therapeutic Protein Product to a Reference Product ^28^ Scientific Considerations in Demonstrating Biosimilarity to a Reference Product ^27^ Biosimilars: Questions and Answers Regarding Implementation of the Biologics Price Competition and Innovation Act of 2009 ^29^ Formal Meetings Between the FDA and Biosimilar Biological Product Sponsors or Applicants ^30^

Summary of three pathways for the development of novel therapeutics that have emerged or have been finalized since 2011.

### Breakthrough therapy designation

The Food and Drug Administration Safety and Innovation Act (FDASIA) was signed on July 9, 2012. FDASIA Section 902 provided the FDA with the ability to establish breakthrough therapy designation (BTD) as a new program within the Expedited Programs for Serious Conditions ^23^. BTD was designed to be available for drugs intended to treat a serious condition and that have been shown to exhibit initial clinical evidence of considerable improvement over pre-existing therapies. The BTD program joined other expedited development and review programs, including fast track designation (1997), accelerated approval (1992), and priority review designation (1992), which have promoted innovation by facilitating the expedited development and review of novel medicines. Since FDASIA was signed in July 2012, CDER has approved 30 original BTDs, one third (10) of which were protein drugs. Over this same period, 26% of CDER-approved biologics (10 out of 39) have been granted BTD designation ( Table 4). Since July 2012, CBER has also approved two drugs under the BTD designation, but neither of these was a recombinant protein.

**Table 4.** Therapeutic proteins granted breakthrough therapy designation upon original submission.

#	Approval date	Drug name (Market name)	Class	Description	Use
1	11/1/2013	Obinutuzumab (Gazyva)	mAb	Humanized anti-CD20	Treatment of patients with previously untreated chronic lymphocytic leukemia in combination with chlorambucil
2	9/4/2014	Pembrolizumab (Keytruda)	mAb	Humanized anti-PD-1	Treatment of patients with unresectable or metastatic melanoma and disease progression following ipilimumab and, if BRAF V600 mutation positive, a BRAF inhibitor
3	12/3/2014	Blinatumomab (Blincyto)	mAb	Mouse bispecific anti- CD19/anti-CD3	Treatment of Philadelphia chromosome- negative relapsed or refractory B-cell precursor acute lymphoblastic leukemia (ALL)
4	12/22/2014	Nivolumab (Opdivo)	mAb	Human anti-PD-1	Treatment of unresectable or metastatic melanoma and disease progression following ipilimumab and, if BRAF V600 mutation positive, a BRAF inhibitor
5	10/16/2015	Idarucizumab (Praxbind)	Fab	Humanized anti- dabigatran	Treatment of patients treated with Pradaxa when reversal of the anticoagulant effects of dabigatran is needed for emergency surgery/urgent procedures and in life-threatening or uncontrolled bleeding
6	10/23/2015	Asfotase-alfa (Strensiq)	Enzyme/fusion protein	Tissue non-specific alkaline phosphatase/Fc fusion/deca-asparatate (D10) peptide	Treatment of patients with perinatal/ infantile- and juvenile-onset hypophosphatasia
7	11/16/2015	Daratumumab (Darzalex)	mAb	Human anti-CD38	Treatment of patients with multiple myeloma who have received at least three prior lines of therapy, including a proteasome inhibitor and an immunomodulatory agent, or are double- refractory to a proteasome inhibitor and an immunomodulatory agent
8	11/30/2015	Elotuzumab (Empliciti)	mAb	Humanized anti- CD319(SLAMF7)	Treatment of patients with multiple myeloma who have received one to three prior therapies
9	12/08/2015	Sebelipase alfa (Kanuma)	Enzyme	Lysosomal acid lipase	Treatment of patients with a diagnosis of lysosomal acid lipase deficiency
10	5/18/2016	Atezolizumab (Tecentriq)	mAb	Humanized anti-PD-L1	Treatment of locally advanced or metastatic urothelial carcinoma who have disease progression during or following platinum-containing chemotherapy or have disease progression within 12 months of neoadjuvant or adjuvant treatment with platinum-containing chemotherapy

Comprehensive listing of all FDA-approved therapeutic proteins granted breakthrough therapy designation upon original submission from July 9, 2012, through August 31, 2016, listed in chronological order of FDA approval. In addition, the class of protein, a brief description, and use are included. BRAF, B-Raf proto-oncogene, serine/threonine kinase; CD, cluster of differentiation; Fab, fragment antigen binding; mAb, monoclonal antibody; PD-1, programmed death receptor-1; PD-L1, programmed death-ligand 1; SLAMF7, SLAM family member 7.

### Orphan designation

Rare diseases substantially impact public health, as an estimated 7,000 different disorders collectively affect approximately 10% of the U.S. population, young children particularly, and many lack effective treatments ^24^. To promote the development of medicines that specifically address unmet medical needs, an orphan designation is given for drugs indicated for the treatment of fewer than 200,000 patients in the U.S. On June 12, 2013, the final regulations amending the 1992 Orphan Drug Regulations were issued ^25^. These amendments clarified and instituted minor changes to regulatory language, such as defining the term “orphan subsets”, the eligibility of designation for previously approved drugs, and the scope of orphan exclusive approval ( https://www.gpo.gov/fdsys/pkg/FR-2013-06-12/pdf/2013-13930.pdf). Under the Orphan Drug Act (ODA) Final Rule, orphan designation grants incentives such as orphan exclusivity for a specific indication (7-year protection from competition), thereby promoting innovation in drug development to help treat patients in greatest need of novel medicines. This program appears to have fulfilled a real need, as 50% of the recently approved therapeutic proteins (31 out of 62) were licensed under the orphan designation ( Table 5).

**Table 5.** Therapeutic proteins granted orphan designation upon original submission (2011–2016).

#	Approval date	Drug name (Market name)	Class	Description	Orphan designation
1	3/25/2011	Ipilimumab (Yervoy)	mAb	Human anti-CTLA-4	Treatment of high-risk stage II, stage III, and stage IV melanoma
2	6/15/2011	Belatacept (Nulojix)	Fusion (Fc)	CTLA-4 Fc-fusion	Prophylaxis of organ rejection in renal allograft recipients
3	11/18/2011	Asparaginase erwinia chrysanthemi (Erwinaze)	Enzyme	Asparaginase erwinia chrysanthemi	Treatment of acute lymphocytic leukemia
4	1/17/2012	Glucarpidase (Voraxaze)	Enzyme	Glucarpidase	Treatment of patients at risk of methotrexate toxicity
5	5/1/2012	Taliglucerase alfa (Elelyso)	Enzyme	Taliglucerase	Treatment of Gaucher’s disease
6	12/14/2012	Raxibacumab (raxibacumab)	mAb	Human anti-anthrax protective antigen (PA)	Treatment of anthrax
7	6/26/2013	Coagulation factor IX recombinant human (Rixubis)	Coagulation factor	Recombinant factor IX	Prophylactic use to prevent or reduce the frequency of bleeding episodes in patients with hemophilia B (routine prophylaxis in patients where there is no evidence or suspicion of bleeding)
8	11/1/2013	Obinutuzumab (Gazyva)	mAb	Humanized anti-CD20	Treatment of chronic lymphocytic leukemia
9	12/23/2013	Coagulation factor XIII A-subunit (recombinant) (Tretten)	Coagulation factor	Recombinant factor XIII A subunit	Prophylaxis of bleeding associated with congential factor XIII deficiency
10	2/14/2014	Elosulfase alfa (Vimizim)	Enzyme	Elosulfase alfa	Treatment of mucopolysaccharidosis type IV A (Morquio A syndrome)
11	2/24/2014	Metreleptin (Myalept)	Hormone	Metreleptin	Treatment of metabolic disorders secondary to lipodystrophy
12	3/28/2014	Coagulation factor IX (recombinant), Fc fusion protein (Alprolix)	Coagulation factor	Recombinant factor IX Fc fusion	Control and prevention of hemorrhagic episodes in patients with hemophilia B (congenital factor IX deficiency or Christmas disease)
13	4/21/2014	Ramucirumab (Cyramza)	mAb	Human anti-VEGFR2 (KDR)	Treatment of gastric cancer
14	4/23/2014	Siltuximab (Sylvant)	mAb	Mouse/human chimeric anti-IL-6	Treatment of Castleman’s disease
15	4/23/2014	Pembrolizumab (Keytruda)	mAb	Humanized anti-PD-1	Treatment of stage IIB through IV malignant melanoma
16	6/6/2014	Antihemophilic factor (recombinant), Fc fusion protein (Eloctate)	Coagulation factor	Recombinant factor VIII Fc-fusion	Treatment of hemophilia A
17	7/16/2014	C1 esterase inhibitor recombinant (Ruconest)	Plasma protein	Recombinant C1 esterase inhibitor	Treatment of (acute attacks of) angioedema caused by hereditary or acquired C1-esterase inhibitor deficiency
18	10/23/2014	Antihemophilic factor porcine, B-domain truncated recombinant (Obizur)	Coagulation factor	Recombinant factor VIII (porcine)	Treatment and prevention of episodic bleeding in patients with inhibitor antibodies to human coagulation factor VIII
19	12/3/2014	Blinatumomab (Blincyto)	mAb	Mouse bispecific anti- CD19/anti-CD3	Treatment of acute lymphocytic leukemia
20	12/22/2014	Nivolumab (Opdivo)	mAb	Human anti-PD-1	Treatment of stage IIb to IV melanoma
21	1/23/2015	Parathyroid hormone (Natpara)	Hormone	Parathyroid hormone	Treatment of hypoparathyroidism
22	3/10/2015	Dinutuximab (Unituxin)	mAb	Mouse/human chimeric anti-GD2	Treatment of neuroblastoma
23	8/27/2015	Evolocumab (Repatha)	mAb	Human anti-proprotein convertase substilisin/ kexin type 9 (PCSK9)	Treatment of homozygous familial hypercholesterolemia
24	10/16/2015	Idarucizumab (Praxbind)	Fab	Humanized anti- dabigatran	To reverse the anticoagulant effect of dabigatran due to uncontrolled life- threatening bleeding requiring urgent intervention or a need to undergo an emergency surgery/urgent invasive procedure
25	10/23/2015	Asfotase-alfa (Strensiq)	Enzyme/fusion protein	Tissue non-specific alkaline phosphatase/ Fc fusion/deca- asparatate (D10) peptide	Treatment of hypophosphatasia
26	11/16/2015	Daratumumab (Darzalex)	mAb	Human anti-CD38	Treatment of multiple myeloma
27	11/24/2015	Necitumumab (Portrazza)	mAb	Human anti-epidermal growth factor receptor	Treatment of squamous non-small cell lung cancer
28	12/8/2015	Sebelipase alfa (Kanuma)	Enzyme	Lysosomal acid lipase	Treatment of lysosomal acid lipase deficiency
29	12/8/2015	von Willebrand Factor (Recombinant) (Vonvendi)	Plasma protein	Recombinant von Willebrand Factor	Treatment of von Willebrand disease
30	3/4/2016	Coagulation factor IX recombinant human (Idelvion)	Coagulation factor	Recombinant factor IX albumin fusion	Treatment of patients with congenital factor IX deficiency (hemophilia B)
31	3/18/2016	Obiltoxaximab (Anthim)	mAb	Mouse/human chimeric anti- *Bacillus anthracis*	Treatment of exposure to *B. anthracis* spores

Comprehensive listing of all FDA-approved therapeutic proteins granted orphan designation upon original submission from January 1, 2011, through August 31, 2016, listed in chronological order of FDA approval. In addition, the class of protein, a brief description, and orphan designation are included. CD, cluster of differentiation; CTLA-4, cytotoxic T-lymphocyte-associated protein 4; Fab, fragment antigen binding; Fc, fragment crystallizable; GD2, disialoganglioside; IL, interleukin; mAb, monoclonal antibody; PD-1, programmed death receptor-1; VEGFR, vascular endothelial growth factor receptor.

### Biosimilars

Therapeutic protein drugs are now a critical component of the overall health-care industry and have revolutionized therapy options in many disease areas. These medications, however, are also some of the most expensive in the marketplace. It had become imperative to extend the generic concept for the licensure of therapeutic protein drugs because, while there existed only a few biopharmaceuticals when the Drug Price Competition and Patent Term Restoration Act of 1984 (Waxman-Hatch Act) was passed, these now account for almost a third of pharmaceutical sales, represent a core component of modern pharmacotherapy, and include the most expensive drugs on the market ^26^. As many biopharmaceuticals are poised to go off-patent, it has been recognized in both the U.S. and Europe that replicating the highly successful model of generic drugs to contain the costs of these therapeutics is a desirable goal, because biosimilar biological products (biosimilars) could potentially reduce the costs of therapeutic protein drugs.

In 2010, as part of the Patient Protection and Affordable Care Act (Affordable Care Act), biologics deemed to be “biosimilar” to an existing FDA-approved reference product were granted an abbreviated drug review and licensure pathway. Over the last few years, the FDA has issued several guidances to help sponsors navigate this novel regulatory pathway ^27– 30^. As of August 31, 2016, three biosimilars have been approved by the FDA (Purple Book ^22^). The first FDA-approved biosimilar, filgrastim-sndz (Zarxio; Sandoz), a biosimilar of filgrastim (Neupogen; Amgen), was approved on March 6, 2015, as a leukocyte growth factor for use in several neutropenia-related indications ^31^. This landmark approval was followed by the approval of infliximab-dyyb (Inflectra; Celltrion), a biosimilar of infliximab (Remicade; Janssen), on April 5, 2016 ^32^, and the approval of etanercept-szzs (Erelzi; Sandoz), a biosimilar of etanercept (Enbrel; Amgen), on August 30, 2016 ^33^; both are tumor necrosis factor (TNF) blockers indicated for use in inflammatory conditions. With the upcoming patent cliff approaching for several “blockbuster” therapeutic protein drugs, there is the expectation that the number of approved biosimilars will increase over the coming years. This trend has already been observed in Europe, where 21 biosimilar medicines have been authorized by the European Medicines Agency since 2006.

Although a drug product is off-patent, competitors do not get direct access to the originator company’s proprietary data or to resources such as the DNA sequence or cell lines used during the manufacture. Typically, the developer of a biosimilar has to retrieve the reference protein as a finished drug product, purify the drug substance, and reverse-engineer the process. Consequently, the FDA biosimilar framework does not require an identical manufacturing process for the innovator product and the biosimilar. Therefore, it is not expected that, in the demonstration of biosimilarity, quality attributes such as protein structure and post-translational modifications measured in comparative physiochemical and functional studies will be identical between the biosimilar and reference product, but highly similar. It has, on the contrary, been argued that deviations from the technology of the innovator may actually be desirable ^9, 34^. This is because since the introduction of the first recombinant DNA-derived therapeutic proteins, the technology to produce and purify these products has greatly improved. An increased focus upon innovation and streamlining of upstream and downstream processing has been reported by the widely respected “Biopharmaceutical benchmarks” series ^3, 35^. Thus, it is not necessary for manufacturers of biosimilars to be locked into the obsolete technologies of the manufacturers of the original products for whom changing methods have major financial and regulatory consequences. With this in mind, it seems much more desirable for biosimilars to be produced and analyzed by the best technology on offer.

## Emerging trends, challenges, and opportunities

As protein-engineering technologies and regulatory frameworks evolve over time, so do protein therapeutics. Optimized versions of existing therapies can be achieved through better drug targeting as well as enhancing potency and functionality. By understanding the mechanism of action as well as the structure-function relationship of a protein, rational design and engineering strategies allow the modification of its activity or the introduction of new activities, leading to customization of existing proteins or the generation of novel therapeutics for specific clinical applications. Here, we will highlight just two examples of rational modifications to existing protein drugs achieved through protein engineering that have led to the approval of novel second-generation therapeutics (see Box 1 and Box 2).

Box 1. **Engineering a second-generation cytotoxic T lymphocyte-associated protein 4 (CTLA4)-Fc fusion**

Abatacept, a CTLA4-Fc fusion protein therapeutic, was developed by Bristol-Myers Squibb and approved by the U.S. Food and Drug Administration (FDA) in December 2005 as the first selective modulator of co-stimulation for the treatment of rheumatoid arthritis ^62^. CTLA4-Fc competitively inhibits CD28 on the surface of T cells for binding to the B7 family co-stimulatory receptors CD80 (B7-1) and CD86 (B7-2) expressed on the surface of antigen-presenting cells. Although this Fc-fusion has been shown to be efficacious in the treatment of rheumatoid arthritis, abatacept was not as effective when tested pre-clinically in non-human primate transplant models ^63, 64^. Experimental data suggested that abatacept did not completely block B7 co-stimulatory receptor-mediated T-cell activation; therefore, a similar molecule with enhanced affinity for CD80/CD86 could be of therapeutic benefit as an immunosuppressant to prevent organ transplant rejection. Belatacept, formerly known as LEA29Y, was therefore developed by Bristol-Myers Squibb as a second-generation CTLA4-Fc. Two amino acid substitutions in the CTLA-4 ligand-binding region (L104E and A29Y) resulted in enhanced *in vitro* binding to CD80 (about two fold more avidly) and CD86 (about four fold more avidly) in addition to greater immunosuppression of T-cell activation *in vitro* (about 10-fold) as compared with the parent molecule (abatacept) ^65^. Belatacept’s enhanced activity was also observed *in vivo* with prolonged renal allograft survival in non-human primates (rhesus monkeys) compared with abatacept ^63, 65^. In clinical studies of kidney allograft recipients, belatacept was shown to be associated with similar levels of patient and graft survival but superior renal function and reduced renal and non-renal toxicities compared with cyclosporine at 12 months after transplant ^66^. The rationally designed analog with enhanced CD80 and CD86 binding, belatacept, a second-generation selective co-stimulation blocker, was approved by the FDA on June 15, 2011, for the prophylaxis of kidney transplant rejection ^67^.

Box 2. **Engineering a second-generation anti-CD20 monoclonal antibody (mAb)**

Rituximab, a chimeric mouse/human type I anti-CD20 mAb, was developed by Genentech and approved by the U.S. Food and Drug Administration (FDA) in November 1997 for the treatment of B-cell non-Hodgkin’s lymphoma. Rituximab binds to CD20 expressed on the surface of many B cells (but not plasma cells), resulting in B-cell depletion via antibody-dependent cellular cytotoxicity (ADCC), complement-mediated cytotoxicity (CMA), and the induction of direct cell death (apoptosis) ^68, 69^. Both ADCC and CMA are dependent upon the Fc region of the mAb interacting with Fc gamma receptor IIIA (FcγRIIIA) or complement component 1q (C1q), respectively. In the case of ADCC, antibody-bound CD20 ^+^ B cells are targeted for cellular depletion by FcγRIIIA-expressing monocytes, macrophages, and natural killer cells. Given the importance of FcγRIIIA engagement and signaling in the mechanism of B-cell depletion, specifically engineering a next-generation mAb ^70– 72^ with enhanced functional activity would be of clinical benefit. Gazyva, formerly known as GA101, a type II anti-CD20 mAb, was engineered and developed by Genentech. This second-generation medicine contains a CD20-binding variable region introduced through protein engineering to take advantage of the potent induction of direct cell death and limited C1q binding typical of type II anti-CD20 antibodies ^51^. The Fc region of this mAb has also been glyco-engineered by producing the protein drug in an expression cell line that overexpresses the glycosylation enzymes β1,4- *N*-acetylglucosaminyltransferase III (GnTIII) and Golgi α-mannosidase II (ManII) ^73^, thereby enriching for afucosylated oligosaccharides. Changes to the Fc glycosylation at Asn297 can lead to changes in FcγR binding, phagocytosis, and cytotoxicity ^74^. In fact, afucosylated antibodies have higher-affinity FcγRIIIA binding and enhanced ADCC activity compared with parent fucosylated counterparts ^20, 75^. The effects of these protein-engineering strategies can be observed in pre-clinical studies as Gazyva demonstrated higher affinity to FcγRIIIA by SPR and induced more potent ADCC when compared with rituximab ^51^. These significant improvements were also observed in phase 3 clinical studies as patients with CD20 ^+^ chronic lymphocytic leukemia treated with Gazyva with chlorambucil had prolonged median progression-free survival time (26.7 months) when compared with patients treated with rituximab with chlorambucil (15.2 months) ^76^. On November 1, 2013, Gazyva became the first glyco-engineered mAb drug to be approved by the FDA. This second-generation anti-CD20 mAb for treatment of chronic lymphocytic leukemia was also the first therapeutic protein to receive breakthrough therapy designation. In addition, Gazyva was granted orphan designation upon approval and contains pharmacogenetics information included on the drug label.

Over this examination of the recently approved therapeutic protein drug landscape, several emerging trends have become apparent. We anticipate that the inclusion of pharmacogenetics information in drug labeling and the importance of “companion diagnostics” will become the focus of increased attention. The FDA has been encouraging drug developers to collect and submit pharmacogenomics data through a guidance, “Pharmacogenomic Data Submissions”, issued in March 2005 ^36^. Pharmacogenetics can play an important role in identifying responders and non-responders to medications, avoiding adverse events, and optimizing drug dose. Drug labeling may contain information on genomic biomarkers and can describe drug exposure and clinical response variability, risk for adverse events, genotype-specific dosing, mechanisms of drug action, and polymorphic drug target and disposition genes. Therefore, pharmacogenetic profiling is of particular importance when potential drug candidates exhibit highly variable safety, efficacy, or pharmacokinetics profiles. In January 2013, the FDA issued a guidance, titled “Clinical Pharmacogenomics: Premarket Evaluation in Early-Phase Clinical Studies and Recommendations for Labeling”, to assist drug developers with conducting exploratory pharmacogenomic investigations, enrichment strategies for clinical trials, adaptive trial designs, or companion diagnostics ^37^. Pharmacogenetic information and changes in drug labeling can lead to drugs targeted for different populations, personalized dosing regimens, and companion diagnostics. There have been 11 therapeutic proteins approved since 2011 that have included pharmacogenetic biomarkers in their drug labels ( Table 6). For a complete listing of drugs with available pharmacogenetics information, see the FDA’s Table of Pharmacogenomic Biomarkers in Drug Labeling ^38^. The FDA’s review process will continue to adapt as the incorporation of pharmacogenetic information becomes more commonplace. This will also require a coordinated cross-center review to incorporate the companion diagnostic/sequencing in the drug development/licensure process.

**Table 6.** Therapeutic proteins with pharmacogenetic biomarkers in drug labeling.

#	Approval date	Drug name (Market name)	Class	Description	Pharmacogenetic biomarker	Therapeutic area
1	6/8/2012	Pertuzumab (Perjeta)	mAb	Humanized anti- human epidermal growth factor receptor 2 (HER2)	HER2 protein overexpression positive	Oncology
2	2/22/2013	Ado-trastuzumab emtansine (Kadcyla)	Antibody- drug conjugate	Humanized anti-HER2/neu conjugated to emtansine	HER2 protein overexpression or gene amplification positive	Oncology
3	11/1/2013	Obinutuzumab (Gazyva)	mAb	Humanized anti-CD20	CD20 antigen positive	Oncology
4	2/14/2014	Elosulfase alfa (Vimizim)	Enzyme	Elosulfase alfa	N-acetylgalactosamine-6- sulfatase deficient	Inborn errors of metabolism
5	9/4/2014	Pembrolizumab (Keytruda)	mAb	Humanized anti-PD-1	(1) BRAF V600 mutation positive, (2) PD-L1 protein expression positive	Oncology
6	12/3/2014	Blinatumomab (Blincyto)	mAb	Mouse bispecific anti-CD19/anti-CD3	Philadelphia chromosome negative	Oncology
7	12/22/2014	Nivolumab (Opdivo)	mAb	Human anti-PD-1	(1) BRAF V600 mutation positive, (2) PD-L1 protein expression positive	Oncology
8	1/23/2015	Parathyroid hormone (Natpara)	Hormone	Parathyroid hormone	Calcium sensing receptor mutation positive	Inborn errors of metabolism
9	3/10/2015	Dinutuximab (Unituxin)	mAb	Mouse/human chimeric anti-GD2	MYCN amplification positive	Oncology
10	7/24/2015	Alirocumab (Praluent)	mAb	Human anti- proprotein convertase substilisin/kexin type 9 (PCSK9)	LDL receptor mutation heterozygotes	Endocrinology
11	8/27/2015	Evolocumab (Repatha)	mAb	Human anti- proprotein convertase substilisin/kexin type 9 (PCSK9)	LDL receptor mutation heterozygotes and homozygotes	Endocrinology

Comprehensive listing of all FDA-approved therapeutic proteins with pharmacogenetics biomarkers in drug labeling from January 1, 2011, through August 31, 2016, listed in chronological order of FDA approval. In addition, the class of protein, a brief description, pharmacogenetics biomarker, and therapeutic area are included. BRAF, B-Raf proto-oncogene, serine/threonine kinase; CD, cluster of differentiation; GD2, disialoganglioside; LDL, low-density lipoprotein; mAb, monoclonal antibody; MYCN, v-myc avian myelocytomatosis viral oncogene neuroblastoma derived homolog; PD-1, programmed death receptor-1; PD-L1, programmed death-ligand 1.

It is reasonable to anticipate that proteins will be more extensively engineered in the future. This means that the new generation of therapeutic proteins will carry neo-sequences not found in nature. Thus, the potential risks of immunogenicity (undesirable immune responses to therapeutic proteins) ^39^ will also increase and, in turn, demand new technologies for immunogenicity risk assessment and mitigation ^39, 40^. Protein engineering is no longer restricted to altering the primary sequence of proteins. On the other hand, the rapidly growing trend of codon optimization involves the substitution of synonymous codons to improve protein synthesis and increase protein production ^41, 42^. A growing scientific literature suggests that although synonymous codons do not alter protein sequence they can have profound effects on protein folding and function ^43– 45^. Consequently, these therapeutic proteins designed by using such strategies will have to be carefully evaluated.

Finally, a confluence of computational and high-throughput experimental methods for protein-engineering and “off the shelf” platform technologies has ushered in unprecedented opportunities to develop safe, effective, and more convenient protein therapeutics. These opportunities do come with risks but rapid advances in new technologies as well as the underlying science suggest that these risks can be managed.
